# A Case of Actinomycosis Hominis

**Published:** 1886-10

**Authors:** A. Schirmer

**Affiliations:** 541 Blue Island Avenue; Chicago


### A CASE OF ACTINOMYCOSIS HOMINIS. *By* DR. A. SCHIRMER, Chicago.

[Read before the Chicago Medical Society.]

Frank P., a Pole, 25 years old, married, is at present employed in a lumber yard. He has been a resident of the United States five years. In Poland he was employed as a farm laborer during the summer, and in winter he attended to barn work. There is no history of any hereditary disease in his family.

When 18 years old, while chopping wood, he received a slight incised wound on the chin. The hæmorrhage from it was slight and no attention was paid to the matter. Shortly afterward some swelling was noticed on both sides of the lower jaw. The swelling on the left side disappeared in a few days, but on the right it remained. Three months after the injury he was obliged to consult a physician on account of the severe pain in the injured part. A clear fluid was injected into the swelling several times, in each instance followed by severe pain. Shortly after the injections the skin turned white, and in a few days opened, discharging a little pus. The fistula remained open and later on the discharge increased. Since this time the patient has been unable to completely open his mouth. Three years ago, on account of a severe cough, he was unable to work for one week, but from that time on he was employed until last May.

In July last, I was called to see the patient at his home. He then complained of severe pains in his left side and stiffness of the neck, so that he was unable to move his head. He was feverish, his pulse was frequent and his temperature was elevated. He had no appetite, and he coughed some. Two days later the fistula was enlarged, its walls dissected out, and some pieces of necrosed bone were removed. At the same time an abscess on the left side of neck, midway between the shoulder and the chin, was opened. It discharged about a tablespoonful of sero-pus, in which floated many sand-like grayish-yellow granules. The opening into the abscess was enlarged and a digital examination proved it to be superficial. The patient could open his mouth better after this operation. Ten days later an abscess on the opposite side of the neck was opened; it discharged about two ounces of pus, somewhat thicker than in the former instance. It also contained granules, but smaller in size than those in the first abscess. Since I first saw him the pain, cough and expectoration have become gradually worse. His appetite has failed, and he does not sleep well. During the last ten days he has complained of severe pains in his right arm.

When I first saw the patient I suspected actinomycosis, and preserved the contents of the abscess. Microscopic examination confirmed my diagnosis, which was concurred in by Professor C. Fenger, who also demonstrated the presence of actinomyces in specimens of the abscess-contents sent him. The fungus has also been observed in the sputum of this patient.

541 Blue Island Avenue.

